# Endoluminal functional lumen imaging probe: a new modality in the evaluation of esophageal disorders in children and preliminary use in eosinophilic esophagitis

**DOI:** 10.3389/fped.2025.1581225

**Published:** 2025-11-18

**Authors:** Jenna Berson, Archana Kota, Jeremiah Levine

**Affiliations:** Division of Pediatric Gastroenterology and Hepatology, New York University School of Medicine, New York, NY, United States

**Keywords:** eosinophilic esophagitis, esophageal motility, distensibility index, FLIP, children

## Abstract

**Introduction:**

Evaluating pediatric esophageal motility and structural disorders such as eosinophilic esophagitis (EoE) remains challenging. Conventional modalities are limited in their ability to assess biomechanical properties of the gastrointestinal tract, such as luminal compliance and distensibility. The endoluminal functional lumen imaging probe (FLIP) is a minimally invasive technology that uses impedance planimetry to measure esophageal parameters. Recent studies have shown the utility of FLIP in quantifying esophageal remodeling and fibrostenotic severity. FLIP-derived esophageal distensibility index (DI) is a sensitive marker for subclinical fibrosis and rigidity, often identifying esophageal narrowing not apparent on endoscopy or by symptom assessment alone. We investigated the use of FLIP to capture early esophageal dysfunction in patients with EoE, especially those in clinical remission.

**Methods:**

Retrospective chart review of patients with EoE who underwent EndoFLIP at our institution. EndoFLIP 2.0 Impedance Planimetry System was used via standard protocol. Variables in patients with EoE were compared by unpaired t-test.

**Results:**

Ten patients fulfilled inclusion criteria and 70% of the cohort were in clinical remission. The average minimum diameter at the narrowest luminal point at the maximum fill volume was 11.3 mm ± 3.3. The average distensibility index was 3.1 mm^2^/mmHg ± 1.4.

**Discussion:**

EndoFLIP was able to detect esophageal dysfunction in pediatric patients with EoE as evidenced by an overall low distensibility index. This adjunctive tool appears particularly useful in asymptomatic patients with EoE in clinical and histologic remission, as it may detect residual esophageal dysfunction highlighting the importance of early detection and ongoing therapy.

## Introduction

Diagnosing and characterizing pediatric esophageal motility and structural disorders such as achalasia, swallowing dysfunction, eosinophilic esophagitis (EoE), and post-surgical stricture remains challenging.

Conventional modalities, including standard endoscopy and radiologic imaging, are limited in their ability to assess key biomechanical properties of the gastrointestinal (GI) tract, such as luminal compliance and distensibility.

The endoluminal functional lumen imaging probe (FLIP) is a minimally invasive technology that uses impedance planimetry to measure luminal diameter, cross-sectional area (CSA), intraluminal pressure, and the derived distensibility index (DI) during controlled balloon distension. It can also detect patterns of contractile activity, including both anterograde and retrograde sequences.

### Technology and equipment

The FLIP system utilizes impedance planimetry to measure CSA within a distension balloon, integrating these data with simultaneous intraballoon pressure recordings (1). The probe incorporates multiple longitudinal electrodes, enabling acquiring CSA at 16 equidistant sites along the balloon (1).

By pooling CSA measurements along the sphincter, FLIP generates a three-dimensional profile of estimated sphincter diameters, offering both geometric and functional characterization (1, 2).

### Methodology and protocol

Placement of the FLIP catheter is typically performed during sedated upper endoscopy or under general anesthesia. The catheter is introduced transorally and positioned so that the balloon spans the esophagogastric junction (EGJ). Occasionally FLIP can be used in other areas on an experimental basis; in those circumstances the catheter may be placed in other regions of interest, such as the pylorus, strictures, or post-surgical sites. Correct positioning is confirmed by identifying the characteristic “waist” of the EGJ on the real-time FLIP display at low balloon fill volumes (20–30 mL). Direct endoscopic visualization can assist placement; however, measurements are ideally obtained without the endoscope in place to avoid altering FLIP metrics (3, 4). The operator manually stabilizes the catheter and may adjust its position during balloon inflation, as esophageal contractions can push the balloon distally.

FLIP is FDA-approved for children 5 years and older and should be used as an adjunct to other diagnostic modalities. There is no standardized pediatric protocol. In clinical practice, balloon volume is titrated carefully according to patient size and indication, with close monitoring to ensure safety. After positioning, the balloon is inflated in 10 mL increments to a target volume of 50–60 mL, guided by intra-balloon pressure or suspected outflow obstruction, with a 30–60 s wait at each volume. The system records cross-sectional area (CSA) and intra-balloon pressure, calculating the DI (DI = CSA ÷ pressure).

### FLIP panometry contractile response: adult classification and pediatric considerations

FLIP panometry contractile response categories are well established in adults based on the Dallas Consensus (5); however, these classifications are not routinely applied in pediatric populations, and currently there are no standardized pediatric norms. Further studies are needed to define normal values and validate clinical relevance in children.

The FLIP contractile response can be divided into five main categories (Table 1): (i) Normal – repetitive antegrade contractions (RACs) or more than one distinct antegrade contraction, typically seen in healthy adults. (ii) Absent—no contractions, observed in severe hypomotility, end-stage scleroderma, or achalasia with aperistalsis. (iii) Spastic—sustained LES or occluding contractions, sometimes with LES lift, seen in type III achalasia, distal esophageal spasm, or hypercontractile (“jackhammer”) esophagus. (iv) Diminished—low-pressure contractions (≤40 mm Hg at 60 mL fill) with few antegrade contractions, seen in hypomotile esophagus or post-surgical cases. (v) Disordered—high-pressure contractions (>40 mm Hg at 60 mL fill) that do not meet spastic criteria, seen in subtle obstruction or early spastic disorders. Other patterns, including repetitive retrograde contractions (RRCs) or vigorous antegrade contractions, are recognized but not formally classified. EGJ opening is assessed using the DI at 60 mL fill. A DI ≥2.8 mm²/mmHg is considered normal, while a DI <2.8 mm²/mmHg is reduced. In adults, the EGJ opening, combined with contractile response categories, informs the overall FLIP motility impression.

**Table 1:** FLIP panometry contractile responses.

Contractile response	Definition	Clinical examples/context
Normal	Repetitive antegrade contractions (RACs) or more than one distinct antegrade contraction	Healthy adults
Absent	No detectable contractions	Severe hypomotility, end-stage scleroderma, achalasia with aperistalsis
Spastic	Sustained LES or occluding contractions, sometimes with LES lift	Type III achalasia, distal esophageal spasm, hypercontractile (“jackhammer”) esophagus
Diminished	Low-pressure contractions (≤40 mmHg at 60 mL fill) with few antegrade contractions	Hypomotile esophagus, post-surgical cases
Disordered	High-pressure contractions (>40 mmHg at 60 mL fill) that do not meet spastic criteria	Subtle obstruction, early spastic disorders
Other patterns	Recognized but not formally classified	Repetitive retrograde contractions (RRCs), vigorous antegrade contractions

### Clinical applications

In pediatrics, FLIP is used to evaluate esophageal motility disorders, guide therapeutic interventions such as myotomy and dilation, assess strictures and congenital anomalies, and monitor remodeling in eosinophilic esophagitis. FLIP is safe and feasible even in young children, making it a valuable adjunct to conventional diagnostic testing.

### Eosinophilic esophagitis (EoE)

EoE represents a chronic, immune/antigen-mediated esophageal disease characterized clinically by symptoms related to esophageal dysfunction and histologically by eosinophil-predominant inflammation (6). The presence of eosinophils in the esophageal mucosa is pathologic (7). Esophagogastroduodenoscopy (EGD) is required to make an accurate, reliable diagnosis when EoE is suspected. Most commonly, 15 eosinophils/hpf (peak value) is the number used to make a diagnosis of EoE. Surface layering, microabscesses, epithelial desquamation, basal zone hyperplasia, and extracellular eosinophil granules have also been noted on histological examinations (8, 9).

Eosinophils are thought to migrate into the esophageal tissue via eotaxin-3, a potent eosinophil chemoattractant which then leads to eosinophil activation and degranulation. Activation of a Th2 immune response results in the production of Th2 cytokines such as IL-13 and IL-4 in response to an allergen. These cytokines stimulate the esophagus to express eotaxin-3. If left untreated, chronic inflammation can promote tissue remodeling and subepithelial fibrosis leading to esophageal rings and ultimately stricture (10). However, esophageal biopsies may not be deep enough to fully capture the extent of subepithelial fibrosis. Early fibrotic remodeling may go undetected yet contribute to esophageal dysfunction (11).

### Application of FLIP in pediatric EoE

Recent clinical studies have shown the utility of FLIP in pediatric EoE, particularly in quantifying esophageal remodeling and fibrostenotic severity. FLIP-derived esophageal distensibility index (DI) is being recognized as a sensitive marker for subclinical fibrosis and rigidity, often identifying esophageal narrowing that is not apparent on endoscopy or by symptom assessment alone (11, 12). FLIP metrics also correlate with composite histologic scores such as the EoE Histology Scoring System, particularly with features of basal zone hyperplasia, dilated intercellular spaces, and eosinophil abscesses, suggesting that FLIP can complement histopathology in assessing disease severity and progression (12).

Current literature suggests that there is reduced distensibility in pediatric patients with EoE compared with controls (13, 14). Typically, distensibility positively correlates with age but in pediatric patients with EoE, esophageal distensibility does not increase with age. It appears that in healthy pediatric patients without EoE, distensibility increases by 0.33 mm per one year of age gained (14). Hoffman et al. (11), found that the DI was significantly lower in patients with fibrotic EoE compared with inflammatory EoE features on endoscopy and did not show a correlation with eosinophil count. They found a DI < 4.5 mm^2^/mmHg predicted grade 2 rings on endoscopy and proposed using a DI < 4.5 mm^2^/mmHg to define esophageal rigidity. In addition to decreased esophageal distensibility in patients with EoE, there is also a notion that esophageal diameter is smaller in patients with EoE. Lynch et al. (15) found that there is an EoE subgroup with abnormal esophageal diameters that lack obvious dysphagia, narrowing, inflammation, or complications. In this population, it is helpful to use an adjunctive tool, such as FLIP, to catch early esophageal dysfunction in patients who appear generally well.

We have investigated the use of FLIP to capture early esophageal dysfunction in patients with EoE, especially those in clinical remission.

## Methods

Retrospective chart review was conducted for all patients who underwent FLIP at our institution from 2022 to 2024. Patients who underwent FLIP but were not ultimately diagnosed with EoE were excluded from data collection. Patient age ranged from 6 to 21 years old. Indications for FLIP use in our EoE population varied from persistent dysphagia, abdominal pain, poor weight gain to asymptomatic patients in clinical remission. All patients had routine screening bloodwork at the time of EGD and FLIP. All patients underwent FLIP at time of EGD using standard protocol adapted from Hoffman et al. (11). Sedation was achieved with propofol with additional agents (including sevoflurane) given at the discretion of pediatric anesthesia. The FLIP 2.0 Impedance Planimetry System was used with an 8-cm or 16-cm catheter based on patient height (8 cm balloon for patient height <120 cm vs. 16-cm balloon for patient height >120 cm). All variables in patients with EoE were compared by unpaired t-test with significance assessed by *P* < 0.05. Study protocol was reviewed and approved by the Institutional Review Board at NYU Grossman School of Medicine.

## Results

Ten patients fulfilled inclusion criteria. Approximately 50% of the cohort underwent FLIP due to persistence of dysphagia. Twenty-percent of the cohort was off all therapy at the time of EGD/ FLIP while the remaining 80% were on therapy, with the majority being on swallowed steroids. None of the cohort were on dupilumab. Thirty-percent of the cohort had grossly normal esophagus, 30% had mixed inflammatory/fibrostenotic features on endoscopy, and 40% had inflammatory features on endoscopy. The majority of cases (70%) were in clinical remission as evidenced by eosinophil count per high-powered field of less than 15, while the remaining 30% had an eosinophil count per high-powered field of >50 with the maximum being greater than 70 eosinophils per high-powered field. Fifty-percent of our cohort had an absolute blood eosinophil count (AEC) of 0.1 cells/microlitre with the remaining 50% having an AEC of >0.5 cells/microlitre (maximum 0.9). The average minimum diameter at the narrowest luminal point at the maximum fill volume was 12.4 mm ± 2.6 (median of 12.9 mm) for the male cohort compared with 9.9 mm ± 4.1 for the female cohort (median of 10 mm, *p* = 0.37). The average minimum diameter for both males and females was 11.3 mm ± 3.3 (median 11.7 mm) (Table 2). The average DI for males and females was 3.1 mm^2^/mmHg ± 1.4 (median of 3 mm^2^/mmHg) (males 3.4 mm^2^/mmHg ± 0.66, females 2.8 mm^2^/mmHg ± 2.3; *p* = 0.58). There was no significant difference in average minimum diameter (*p* = 0.28) and DI (*p* = 0.77) between those children who had active eosinophilic inflammation on biopsies compared with those who were in clinical remission. Similarly, there was no significant difference in average minimum diameter (*p* = 0.12) and DI (*p* = 0.45) between those who had AEC > 0.5 compared with those with normal AEC (Table 3).

**Table 2:** EndoFLIP measurements.

EndoFLIP measurements	Males (*n* = 7)	Females (*n* = 3)	Total (*n* = 10)
Minimum diameter (mm)	12.4 ± 2.6 (12.9)	9.9 ± 4.1 (10, *p* = 0.37)	11.3 ± 3.3 (11.7)
Distensibility index (mm^2^/mmHg)	3.4 ± 0.66 (3.0)	2.8 ± 2.3 (3, *p* = 0.58)	3.1 ± 1.4 (3)

Mean ± SD (median).

**Table 3:** Minimum diameter and DI compared with Eos/HPF and AEC.

Eos/HPF and AEC	Minimum diameter (mm)	Distensibility index (mm^2^/mmHg)
Maximum eosinophils/hpf		
0–14	10.8 ± 3.2 (10.9)	3.1 ± 1.5 (3)
>15	14.9 (*p* = 0.28)	3.6 (*p* = 0.77)
Absolute eosinophil count (AEC)		
0.1–0.4	10.1 ± 3.1 (10)	2.9 ± 1.6 (3)
0.5–0.9	14.5 ± 0.64 (14.5, *p* = 0.12)	3.7 ± 0.75 (3.6, *p* = 0.45)

Mean ± SD (median).

## Discussion

At our institution, FLIP has been used as an adjunctive tool in children with EoE undergoing follow-up EGD. Although our sample size is small, it seems that esophageal dysfunction was detected in patients with EoE as evidenced by an overall low average DI of 3.1 mm^2^/mmHg ± 1.4, using a DI of less than 4.5 mm^2^/mmHg to constitute abnormal distensibility (11). As discussed, the primary management outcome in EoE is histology, but EoE is a patchy disease and esophageal eosinophilia may not correlate with symptoms (12) nor is the degree of fibrosis with or without esophageal dysfunction fully assessed with standard esophageal biopsies. To this point, 30% of our cohort were asymptomatic and 70% of our cohort were in histological remission, yet the DI's were low ranging from 3 to 5 mm^2^/mmHg. In contrast, our patients with persistent EoE with >50 eosinophils per high powered field also had a low DI of 3.6 mm^2^/mmHg. This observation supports the findings of Hoffman et al. (11) that histologic eosinophil count does not correlate with DI. We found no statistically significant difference in minimum diameter and DI between children with active eosinophilic inflammation on biopsies compared with those who were in clinical remission nor a difference in average minimum diameter and DI between children with an AEC > 0.5 compared with those with normal AEC. Although AEC is not used for monitoring or treatment response in EoE, the literature suggests that active EoE status seems to correlate with higher AEC when compared to quiescent EoE status (16, 17). The lack of difference between active EoE and remission EoE is not conclusive since the sample number in our study was small. Our study also supports the findings of Lynch et al. (15) that patients with EoE, especially those who lack obvious dysphagia, narrowing, inflammation, or complications have abnormal esophageal diameters. Again, 30% of our cohort were asymptomatic and 70% of our cohort were in histological remission, but the average esophageal diameter was only 11.3 mm ± 3.3 compared to the expected esophageal diameter of 14 mm in children >6 years old.

## Conclusions

Although FLIP provides valuable insights into pediatric esophageal disease, its use is limited by a lack of pediatric normative data, variable protocols, interoperative variability, device size constraints—particularly in very young or small children, although studies suggest feasibility and safety in those under five years (18) —and limited availability and expertise. Additionally, our small sample size precludes making recommendations regarding use of FLIP in all patients with EoE. However, FLIP seems to detect esophageal dysfunction in pediatric patients with EoE. This adjunctive tool appears particularly useful in asymptomatic patients with EoE in clinical and histologic remission, as it may detect residual esophageal dysfunction highlighting the importance of early detection and ongoing therapy. The full utility and efficacy of FLIP use in the pediatric population is still ongoing and necessary as FLIP bears the potential of acting as the first standardized functional parameter in EoE. More studies with larger sample sizes are needed in addition to standardization of FLIP use and its measurements.

## Data Availability

The raw data supporting the conclusions of this article will be made available by the authors, without undue reservation.
